# American Public Health Association

**Published:** 1879-11

**Authors:** J. L. Cabell

**Affiliations:** President American Public Health Association, and Chairman of Executive Committee; University of Virginia, Charlottesville


AMERICAN PUBLIC HEALTH ASSOCIATION.—The next annual meeting of this important association will he held in Nashville, Tenn., commencing on the 18th of November and closing on the 21st. We copy the following announcement in regard to the chief topics that will engage the attention of the association at that meeting, for the information of our readers:

ANNOUNCEMENT.

The executive committee of the American Public Health Association has decided to supplement the discussion on certain points relating to CITY SANITATION heretofore ordered for the Nashville meeting and announced in the president's circular of August 15, by one on the practical questions connected with the management of an actual or threatened outbreak of yellow fever. It is considered proper that the whole country should have the benefit of the practical lessons taught by the epidemic visitations of 1378 and 1879, and it is fit that the popular diffusion of this knowledge should be made through the medium of this association, which will have an unusually favorable opportunity at the meeting in Nashville, November 18–21, for collecting and recording the conclusions of intelligent and skilled observers as to the practical working of the measures recently put into operation by State and municipal authorities, aided by the national board of health, with a view to prevent the spread of the disease from local sources of infection.

The oral discussion will be prefaced by the reading of several papers by members of the association who have been actively engaged in this practical work during the prevalence of the existing epidemic in Memphis and elsewhere. In order to give definite direction to the discussion the executive committee has adopted the following schedule of the points to be especially considered:

1. How to deal with a city in the yellow fever zone in order to prevent the appearance of a first case.

2. How to prevent the importation of a first case.

3. How to deal with a first case and early cases generally when, in spite of precautions under first and second headings, it has made its appearance.

4. The duty of local boards of health, or other health authorities to report such cases promptly, even though there may be some doubt as to the diagnosis. Whether the knowledge that such reports would be faithfully made would not have a tendency to allay apprehensions and give confidence to other communities while warning them of the importance of making preparations for contingencies.

5. Under what circumstances may it become necessary or expedient to remove the unacclimated portion of the population from an infected place? How may this be effected for the poorer classes of the population, and how should the people thus removed be cared for and supported?

6. Measures for isolating a dangerously infected place.

7. Organizations for the relief and treatment of the sick in an infected city.

8. Measures for preventing the spread of the disease from an infected place by railroads, including the management of transfer stations.

9. Inspection of steamboats at an infected place and at intermediate stations between the port of departure and their final destination. Should stations of observation be established by the national board of health? If so, what should be their relations to the health authorities of the States within whose territorial limits they may be established?

10. Results of the co-operation and aid given by the national board of health to State and municipal boards under the provisions of the act approved June 2, 1879. What suggestions may be made to render this system more efficient?

J. L. CABELL,
*President American Public Health Association.*

The following is the circular above referred to:

*To the Members of the American Public Health Association:*

GENTLEMEN: At a meeting of the executive committee, held in Washington, January 3, 1879, it was decided that the principal subject for discussion at the next annual meeting of the association, to be held in Nashville, Tenn., 18th–21st November, shall be the sanitary condition of cities and towns, especially those of the Southern States.

In selecting a subject of so wide a scope the committee considers that it will be expedient, if not indeed indispensable to the attainment of useful results, to limit the inquiry to certain specified branches of the general subject rather than to attempt to cover the entire ground of city sanitation.

A few years ago a committee appointed by the association prepared a series of elaborate schedules of questions for facilitating such an inquiry, which have been recently published in pamphlet form as circular No. 2, of the national board of health. A copy of this pamphlet will be furnished to any member of the association who desires to take a part in the proposed discussion, on application to Dr. T. J. Turner, secretary of the national board of health, and a member of the executive committee of this association. His address is, "Office National Board of Health, Washington, D. C."

The executive committee recommends the following subjects of inquiry: Water supply, schedule C; drainage and sewerage, schedule D; disposal of garbage and excreta, schedule H; slaughter-houses and abattoirs, schedule K; public-school buildings, schedule M; public-health laws, regulations, etc., schedule R; expenses of municipal sanitation, schedule U.

Attention is also called to the following resolution, presented by Dr. A. L. Gihon, U. S. N., at the last annual meeting:

"*Resolved*, That the executive committee be directed to provide for the investigation and discussion, at the next annual meeting, of the most effective means for preventing the spread of venereal disease."

Members who propose to consider these subjects, or who may desire to treat exhaustively some special subject of their own selection, are invited to prepare papers not to require more than thirty minutes in reading, and to forward titles and abstracts in accordance with section 8 of the constitution.

J. L. CABELL,
*President American Public Health Association,*
*and Chairman of Executive Committee.*

UNIVERSITY OF VIRGINIA,
*Charlottesville, August* 15, 1879.

